# Genotype characterization and delayed loss of ambulation by glucocorticoids in a large cohort of patients with Duchenne muscular dystrophy

**DOI:** 10.1186/s13023-021-01837-x

**Published:** 2021-04-28

**Authors:** Shu Zhang, Dongdong Qin, Liwen Wu, Man Li, Lifang Song, Cuijie Wei, Chunling Lu, Xiaoli Zhang, Siqi Hong, Mingming Ma, Shiwen Wu, National DMD Research Network of “One City, One Doctor”

**Affiliations:** 1 Department of Neurology, First Medical Center of Chinese PLA General Hospital, Beijing, 100853 China; 2 Department of Neurology, Third Medical Center of Chinese PLA General Hospital, Beijing, 100039 China; 3 Department of Physiology, Yunnan University of Chinese Medicine, Kunming, 650500 Yunnan Province China; 4 Department of Neurology, Hunan Children’s Hospital, Changsha, 410008 Hunan Province China; 5 Department of Neurology, The Second Hospital of Shanxi Medical University, Taiyuan, 030001 Shanxi Province China; 6 Department of Pediatric Neurology, Henan Children’s Hospital, Zhengzhou, 450018 Henan Province China; 7 Department of Pediatrics, Peking University First Hospital, Beijing, 100034 China; 8 Department of Muscle Atrophy, Affiliated Yiling Hospital of Hebei Medical University, Shijiazhuang, 050091 Hebei Province China; 9 Department of Pediatrics, The Third Affiliated Hospital of Zhengzhou University, Zhengzhou, 450052 Henan Province China; 10 Department of Pediatrics, Chongqing Medical University Affiliated Children’s Hospital, Chongqing, 400042 China; 11 Department of Neurology, Affiliated People’s Hospital of Zhengzhou University, Zhengzhou, 450003 Henan Province China; 12 Department of Neurology, Chinese PLA General Hospital, 28 Fuxing Road, Haidian District, Beijing, 100853 China

**Keywords:** Duchenne muscular dystrophy, Genotype, Phenotype, Glucocorticoid treatment

## Abstract

**Background:**

Duchenne muscular dystrophy (DMD) is the most common genetic muscle disease in human. We aimed to describe the genotype distribution in a large cohort of Chinese DMD patients and their delayed loss of ambulation by glucocorticoid (GC) treatments. This is to facilitate protocol designs and outcome measures for the emerging DMD clinical trials.

**Results:**

A total of 1163 patients with DMD were recruited and genotyped. Genotype variations were categorized as large deletions, large duplications, and small mutations. Large deletions were further analyzed for those amenable to exon-skipping therapies. Participants aged 5 years or older were grouped into GC-treated and GC-naïve groups. Clinical progression among different genotypes and their responses to GC treatments were measured by age at loss of ambulation (LOA). Among the mutation genotypes, large deletions, large duplications, and small mutations accounted for 68.79%, 7.14%, and 24.07%, respectively. The mean age at diagnosis was 4.59 years; the median ages at LOA for the GC-naïve, prednisone/prednisolone-treated, and deflazacort-treated groups were 10.23, 12.02, and 13.95 years, respectively. The “deletion amenable to skipping exon 44” subgroup and the nonsense-mutation subgroup had older ages at LOA than the “other deletions” subgroup. Subgroups were further analyzed by both genotypes and GC status. All genotypes showed significant beneficial responses to GC treatment. Deletions amenable to skipping exon 44 showed a lower hazard ratio (0.155). The mean age at death was 18.57 years in this DMD group.

**Conclusion:**

Genotype variation influences clinical progression in certain DMD groups. Beneficial responses to GC treatment were observed among all DMD genotypes. Compared with other genotypes, deletions amenable to skipping exon 44 had a lower hazard ratio, which may indicate a stronger protective effect of GC treatments on this subgroup. These data are valuable for designing future clinical trials, as clinical outcomes may be influenced by the genotypes.

## Background

Duchenne muscular dystrophy (DMD) is a genetic neuromuscular disorder caused by mutations in the dystrophin gene. Clinical symptoms of DMD include progressive muscle weakness in early childhood resulting in the loss of independent ambulation (LOA) before the age of 12 [1]. Often, in Becker muscular dystrophy (BMD), which is the mild allelic form with in-frame mutations of the dystrophin gene, LOA occurs after the age of 16 [1]. With the increased availability of the standard of care and glucocorticoid (GC) treatments, the average age at LOA in DMD has been delayed until after the age of 14 [2].

The recent discoveries of new therapeutic approaches to DMD have resulted in several clinical trials being conducted globally, participated by Chinese patients. We built a national registry in collaboration with the Treatment of Neuromuscular Diseases (TREAT-NMD) Network in 2012 (www.dmd-registry.com) and reported the initial genetic characterization of dystrophinopathies in China [3].

In this study, we recruited a large cohort and conducted more detailed genetic analyses on patients from nine neuromuscular centers in China. We performed detailed clinical studies of different genotypes, including those that are currently, or predicted to be, amenable to exon-skipping therapies. We compared the ages at LOA and the duration of survival to provide data for designing future clinical trials.

## Results

### Patient ascertainment and enrollment

**Fig. 1:**
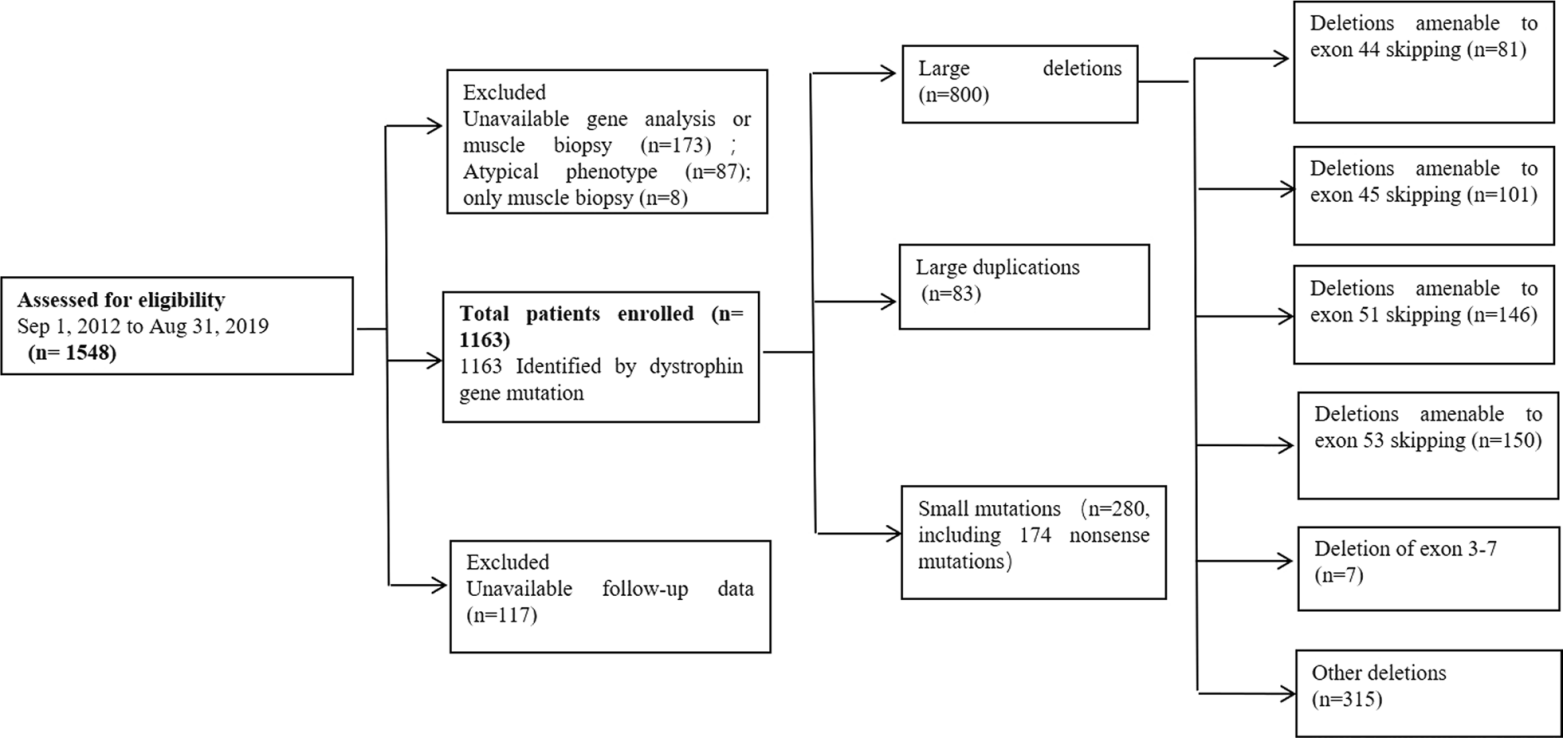
Participant ascertainment and genotype distribution

The enrollment process of this study and the patients’ genotype distribution are described in Fig. 1. We initially recruited 1548 Chinese patients with DMD. Among these participants, accurate genetic analysis results to confirm the diagnosis of DMD were not available for 173 patients, 8 patients were diagnosed using clinical features and muscle biopsy results without genetic analyses, 87 showed atypical phenotypes, and 117 had no valid follow-up data. All these patients were excluded from the study.

Statistical analyses were conducted on the data collected from the remaining 1163 participants who were genetically and clinically diagnosed. The mean age of the 1163 participants was 8.9 years (range, 0.1 to 33.7 years). All 1163 participants were male of Chinese descent. A total of 104 patients were lost to follow-up. Data analysis was based on the last valid follow-up data.

### Genotype distribution of dystrophin gene mutations

Among all mutations, large deletions, large duplications, and small mutations accounted for 68.79% (800/1163), 7.14% (83/1163), and 24.07% (280/1163), respectively (Fig. 1). One patient had a combination of exon 51 deletion and exon 64–79 duplication. Of the 280 small mutations, 174 were nonsense mutations. We further divided large deletions into those with exon 3–7 deletion, those that were amenable to exon-skipping therapies (exons 44, 45, 51, and 53), and the “other deletions” subgroup, which included those deletions that could not be assigned to the aforementioned specific exon deletion patterns and were later used as the reference group for comparisons of clinical progression and responses to GC treatments. The detailed deletion patterns and the number of patients amenable to current and potential exon-skipping therapies are provided in Table 1. The patient with a single exon 52 deletion, amenable to skipping both exon 51 and exon 53, was assigned to the deletions amenable to exon 53 skipping subgroup.

**Table 1:** Number of patients with exon deletions amenable to exon-skipping therapy

Exons to be Skipped	Exons deleted (number of patients)	Number of patients (Total)
44	Exons 17–43 (2); exons 19–43 (2); exons 35–43 (1); exons 38–43 (1); exons 40–43 (2); exon 41–43 (1); exon 43 (7); exon 45 (51); exons 45–54 (16); exons 45–56 (1)	81
45	Exons 12–44 (1); exons 18–44 (3); exon 44 (26); exon 46 (1); exons 46–47(28); exons 46–48 (13); exons 46–49 (5); exons 46–51 (16); exons 46–53 (3); exons 46–55 (3); exons 46–57 (2)	101
51	Exons 3–50 (1); exons 17–50 (1); exons 30–50 (1); exons 35–50 (1); exons 45–50 (44); exons 47–50 (5); exons 48–50 (38); exons 49–50 (37); exon 50 (18)	146
53	Exons 45–52 (48); exons 47–52 (6); exons 48–52 (36); exons 49–52 (30); exons 50–52 (8); exon 52 (22)^a^	150

^a^Patient with a single exon deletion of exon 52, amenable to both exon 51 and 53 skipping, was assigned into the “deletions amenable to exon 53 skipping” subgroup in this study

### Clinical progression of patients with different DMD genotypes

#### Age at diagnosis

The mean age at diagnosis was 4.59 years for the entire group of patients with DMD. Patients with nonsense mutations were older (5.03 years) at diagnosis than those in the “other deletions” subgroup (4.36 years), maybe suggesting a milder phenotype. No significant difference was observed among the other mutation subgroups compared with the “other deletions” subgroup. Age at diagnosis for those in the “exon 44 amenable skipping” subgroup was 4.68 years, whereas the ages of those in the “deletions amenable to skipping exon 45, skipping exon 51, and skipping exon 53” subgroups were 4.84 years, 4.41 years, and 4.35 years, respectively. Interestingly, we found that patients tended to be diagnosed earlier the more recently they were born. Approximately 48% of the patients in this study who were diagnosed before the age of 3 years had high CK and/or elevated transaminases during their routine medical examination for kindergarten enrollment, which is a widespread practice in China. In view of the recent availability of molecular diagnostic tools, we further analyzed the age at diagnosis of patients before and after 2010. In this study, 550 patients were born before 2010, and their mean age at diagnosis was 5.7 years. The other 613 patients born in or after 2010 had an average age of 3.5 years at diagnosis.

#### Age at LOA among the different Duchenne muscular dystrophy genotypes

**Fig. 2:**
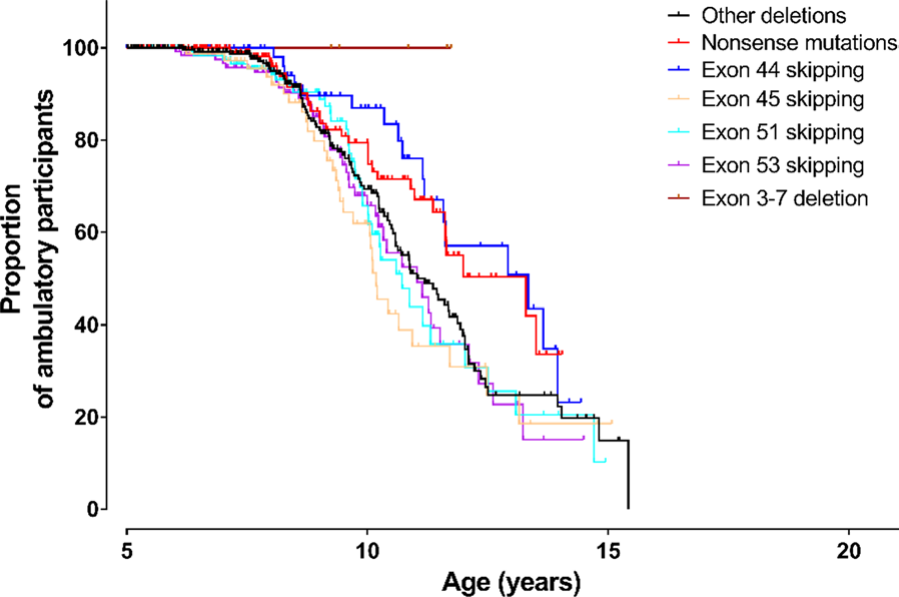
Age at LOA in the different DMD mutation subgroups. The black line indicates the “other deletions” subgroup with deletions not amenable to skipping of exons 44, 45, 51, or 53, as well as exon 3–7 deletion. The red line indicates the nonsense-mutation subgroup. The blue line indicates the “deletions amenable to skipping of exon 44” subgroup. The Navajo white line indicates the “deletions amenable to skipping of exon 45” subgroup. The cyan line refers to the “deletions amenable to skipping of exon 51” subgroup. The purple line indicates the “deletions amenable to skipping of exon 53” subgroup. The brown line indicates the exon 3–7 deletion subgroup. The “other deletions” subgroup was the reference group, and participants with deletions amenable to skipping of exon 44, exon 45, exon 51, exon 53, deletion of exon 3–7, and nonsense mutations were compared with the reference group. All participants in each mutation group were included. *LOA* loss of ambulation, *DMD* Duchenne muscular dystrophy

During the study period, 290 patients experienced LOA. The number of participants with LOA in each mutation subgroup and the median age at LOA are shown in Table 2. Kaplan–Meier plots of the mutation groups are shown in Fig. 2. The “other deletions” subgroup had a median age at LOA of 11.03 years (n = 315; 95% CI, 10.29 to 11.77 years). Patients with deletions amenable to skipping exon 44 and nonsense mutations were significant older at LOA than those in the “other deletions” subgroup (p = 0.029 and p = 0.045, respectively). Subgroups with younger ages at LOA (but not statistically significant) included the “deletions amenable to skipping exon 45, exon 51, and exon 53” subgroups (Table 2). All eight exon 3–7 deletion patients had maintained the independent ability to ambulate at the last follow-up.

**Table 2:** Participant distribution, median age at LOA, and Cox regression

Cox regression factor	Level of factor	Total No. of participants (No. with LOA)	Median age, years, at LOA (95% CI)	HR (95% CI)	p Value
*DMD* mutation	Other deletions	315 (92)	11.03 (10.29, 11.77)	1^b^	–
	Nonsense mutations	174 (31)	13.29 (11.24, 15.34)	0.66 (0.44, 0.99)	0.045^a^
	Exon 44 amenable skipping	81 (18)	13.34 (10.53, 16.15)	0.56 (0.33, 0.94)	0.029^a^
	Exon 45 amenable skipping	101 (31)	10.18 (9.79, 10.57)	1.31 (0.87, 1.97)	0.193
	Exon 51 amenable skipping	146 (31)	10.72 (9.90, 11.54)	1.08 (0.72, 1.63)	0.709
	Exon 53 amenable skipping	150 (40)	11.03 (10.06, 12.00)	1.13 (0.78, 1.65)	0.508
GC drug	Untreated (or treated < 1 month)	326 (111)	10.23 (9.97, 10.49)	1^b^	–
	Deflazacort	65 (4)	13.95 (11.80, 16.10)	0.06 (0.02, 0.19)	< 0.001
	Prednisone or prednisolone	465 (146)	12.02 (11.40, 12.64)	0.40 (0.31, 0.52)	< 0.001

CI, confidence interval; DMD, dystrophin gene; HR, hazard ratio; LOA, loss of ambulation

^a^Significant

^b^An HR of 1 is assigned to factor levels that are taken as reference in the Cox regression model

#### Mean age at death

Among all patients, 22 died during the study period. The mean age at death in this group of patients was 18.57 years (range, 13.3–33.4 years). Eleven deceased patients had experienced a pulmonary infection and died of respiratory failure, six patients died of cardiac failure, and five patients died of unknown causes. Among the 290 non-ambulatory patients in our study, only two used intermittent nocturnally assisted ventilation.

Since the participants of this cohort were much younger than those in previous studies, the number of patients who died during the study was quite small; the length of survival and Kaplan–Meier plots of different mutation groups were not further analyzed.

### Differential response to GC treatment

#### Treatment with different GCs

Of the 979 participants older than 5 years, 530 received some form of GC treatment, 123 received an intermittent period, and 326 were GC-naïve. Among the 653 patients with known GC exposure, 71 were switched from prednisone/prednisolone to deflazacort. The most common doses were 0.9 mg/kg/day for deflazacort and 0.3 to 0.75 mg/kg/day for prednisone/prednisolone daily. Four patients received 1.5 mg/kg dosing (10 days “on” and 20 days “off”) of prednisone/prednisolone, and nine patients were administered a dose of 0.75 mg/kg/day every other day in this study. The most common side effects were cushingoid features, weight gain, growth delay, cataract, intraocular hypertension, and behavioral changes as previously reported [3].

#### Clinical responses to glucocorticoid treatment among the different genotypes

Among all participants aged 5 years or older, the median age at LOA was 10.23 years for the GC-naïve group, 12.02 years for patients treated with prednisone/prednisolone (hazard ratio [HR] = 0.40, p < 0.001), and 13.95 years for patients treated with deflazacort (HR = 0.06, p < 0.001). Both GC-treated groups displayed later LOA compared to the GC-naïve group (Table 2).

We further compared the responses to GC treatment among the different mutation subgroups (Table 3). A significantly older median age at LOA was observed among all mutation subgroups. An HR of 1 was assigned to GC treatment for less than 1 month, as it was used as a reference in the Cox regression model, and the p values in Table 3 represent the comparisons between the GC-treated versus GC-untreated patients in each row. Deletions amenable to skipping exon 44 showed a lower HR (0.155). Patients with deletions amenable to skipping of exons 45, 51, and 53, who seemed to have more severe phenotypes in this study, demonstrated a different response to GC treatment with a higher HR (Table 3).

**Table 3:** Effects of glucocorticoid treatment on median age at loss of ambulation in the different genotypes

*DMD* mutation cox	GC treatment for less than 1 month or never			Continuous GC treatment for 12 months or longer			
	Total No. of participants (No. with LOA)	Median age, years, at LOA (95% CI)	HR ^a^	Total No. of participant (No. with LOA)	Median age, years, at LOA (95% CI)	p Value	HR (95% CI)
Other deletions	139 (36)	10.530 (10.177–10.883)	1	142 (46)	12.090 (11.642–12.538)	0.0003^b^	0.436 (0.277–0.685)
Nonsense mutations	73 (12)	10.900 (9.293–12.507)	1	84 (19)	13.290 (11.482–15.098)	0.024^b^	0.418 (0.196–0.891)
Exon 44 amenable skipping	32 (6)	11.580 (9.189–13.971)	1	36 (9)	13.650 (12.987–14.313)	0.003^b^	0.155 (0.045–0.535)
Exon 45 amenable skipping	42 (13)	10.100 (8.883–11.317)	1	46 (14)	11.710 (9.292–14.128)	0.007^b^	0.327 (0.145–0.739)
Exon 51 amenable skipping	65 (11)	10.010 (9.372–10.648)	1	63 (18)	11.150 (9.82–12.318)	0.032^b^	0.419 (0.189–0.927)
Exon 53 amenable skipping	51 (14)	10.000 (9.221–10.779)	1	81 (21)	11.510 (10.514–12.506)	0.001^b^	0.266 (0.125–0.566)

*CI*  confidence interval, *DMD* dystrophin gene, *GC* glucocorticoid corticosteroid, *HR *hazard ratio, *LOA* loss of ambulation

^a^An HR of 1 is assigned to the GC treatment for less than 1 month or never, as it is used as a reference in the Cox regression model

^b^Significant (the p value represents the comparison of GC-treated versus GC-untreated patients in each row)

## Discussion

Natural history plays a paramount role in rare disease clinical trials and drug development. The United States Food and Drug Administration recommended that in single-arm interventional trials for rare diseases, natural history studies should be used as an external control [4]. In the current study, we described the genotype distribution of a cohort of Chinese patients with DMD and their responses to GC treatment. The clinical progression of the different genotype groups was measured by age at LOA.

Although detecting disease onset is an important part of the natural history of DMD, measuring the precise age at symptom onset is difficult because the delay in motor development is insidious and the symptoms progress slowly during the first few years of life. Approximately half the patients in this study who were diagnosed before the age of 3 years had high CK and/or elevated transaminases during their routine medical examination for kindergarten enrollment, which is a widespread practice in China. The average age at diagnosis of DMD is approximately 4 to 5 years worldwide [5–7]. The mean age at diagnosis was 4.59 years in our study, which is similar to the age of 4.43 years reported by the Parent Project Muscular Dystrophy [6]. The mean age at diagnosis in Italy was 3.4 years overall [8] and 3.5 years in patients born in or after 2010 in our study. These data indicated that patients born more recently tend to be diagnosed earlier, possibly because of the increased awareness of DMD and the availability of diagnostic tools. Several provinces in China have now included DMD in newborn screening to provide earlier diagnosis [9].

The distribution of DMD genotypes is similar worldwide. Our study showed that 75.9% of the probands had out-of-frame large deletions and duplications (multi-exon or single exon) and 24.1% had small mutations. These data corroborated the results of the TREAT-NMD DMD Global Database (80% large mutations and 20% small mutations) [10], which agrees with our previously finding [3]. The proportion of small mutations was slightly higher in this study, which could have been caused by the increased recruitment efforts to implement an ongoing clinical trial in China (PTC124-GD-041-DMD) studying patients with nonsense mutations in the dystrophin gene.

The benefits of GC treatment shown on the time function test, muscle strength, and forced vital capacity of patients with DMD were first reported in 1974 [11]. Further studies have confirmed the advantage of this treatment [12], and it has been recommended as a part of the standard care for DMD [13, 14]. As previously reported, treatment with deflazacort delayed the age at LOA by 1 year compared with treatment with prednisone/prednisolone [2, 15]. In the current study, we observed a nearly 2-year delay in LOA in the deflazacort group compared with the prednisone/prednisolone group. This is similar to the results of the NorthStar Database [16, 17].

Correlations between age at LOA and DMD genotype subgroups have been reported by previous studies [18, 19]. Exon 3–7 and single exon 45 deletions (amenable to exon 8 and exon 44 skipping) maintained longer ambulation than the other mutation subgroups. The milder phenotype was proposed to be due to endogenous exon skipping resulting in retaining partial dystrophin [20–22]. In our study, the age at LOA for those with deletions eligible for exon 44 skipping and those with nonsense mutations was significantly greater than that of patients in the “other deletions” subgroup. No patient with exon 3–7 deletion lost independent ambulation during the study period. Patients in the “deletions amenable to exon 45, 51, and 53 skipping” subgroups were younger at LOA, as also previously reported [23]. Therefore, genotype differences clearly influenced the clinical progression of DMD. Three mutation subgroups, i.e., the “nonsense mutations,” “exons 3–7 deletion,” and “deletions amenable to exon 44 skipping” had significantly milder clinical progression than the other subgroups. Some of these patients had such mild phenotype that they were excluded from the current study because of their “atypical” phenotype at the initial recruitment.

GC treatment has been proven beneficial for DMD. Our current study showed that all genotype subgroups benefited from GC treatment by significantly delaying LOA. Treatment with deflazacort appears to be more beneficial in delaying LOA compared with prednisone in all genotypes. However, it is possible that different mutation subgroups could show differential HR to GC treatment. Compared with other genotypes, deletions amenable to skipping exon 44 had a lower hazard ratio, which may indicate a stronger protective effect of GC treatments on this subgroup. Subgroups with severe phenotypes, such as the “deletions amenable to skipping of exon 45 and exon 51” subgroups, had a higher HR than the “deletions amenable to skipping of exon 44” regarding the response to GC treatments, which might be due to the rapid disease progression from an early age and the lack of sufficient time for the GC treatment to work. However, more evidence is needed to confirm this speculation.

With improvements in medical technology and the implementation of the standard of care, most DMD individuals are now living into their 30s and even 40s in Japan and some European countries [14, 24, 25]. Multidisciplinary intervention, including pulmonary, orthopedic, cardiac, and rehabilitation care, is currently the most effective way to enable long-term survival for patients with DMD. Currently, cultural and economic barriers have prevented parents from accepting the use of a ventilator for patients with DMD in China. Among the 290 non-ambulatory patients in our study, only two patients used intermittent nocturnal assisted ventilation. This reluctance to use assisted ventilation, coupled with the systemic difficulty in implementing the standard of care, may have contributed to the shorter survival of our study cohort. Since 2015, we have initiated a “one city, one doctor” project to promote awareness and implement the standard of care for patients with DMD in China [26]. We have seen improvements in the long-term care and survival of this group of patients.

## Conclusion

In summary, this study provides detailed genotype characterization of a large cohort of Chinese patients with DMD. Detailed clinical progression within each genotype was studied by measuring age at diagnosis, age at LOA, and length of survival. We identified significant differences in clinical progression among the different DMD genotypes. The uniform positive responses to GCs confirmed the benefits of these treatments regardless of the DMD genotypes. However, the use of deflazacort clearly conferred larger clinical benefits than the use of prednisone. These data constitute an important knowledge base for designing future clinical trial protocols, as patients’ clinical outcomes may be influenced by their DMD genotypes.

## Methods

### Protocol approvals, registrations, and consent

This study was approved by our institutional ethics committee (No. 2015003). Nine major neuromuscular centers participated in this study (see author affiliations). Participants were recruited between March 1, 2015, and August 31, 2019. They were enrolled and followed up every year. Written or online informed consent was obtained from patients or their legal guardians. All data were entered and analyzed anonymously.

### Recruitment criteria

Inclusion criteria were confirmed DMD diagnosis by genetic analysis and no participation in any clinical trial. The patients had to have shown phenotypic evidence of DMD before the age of 5, including progressive muscle weakness (proximal > distal), Gowers' sign, calf pseudohypertrophy, characteristic waddling gait, and elevated serum creatine kinase (CK).

Two groups of patients were excluded from this study since they had atypical clinical phenotypes, i.e., those who did not receive GC treatment and were able to maintain independent ambulation beyond the age of 12 and those who received GC treatment and were ambulatory beyond the age of 16. Participants who did not have valid follow-up data or could not be followed up for more than one year were also excluded.

### Genotype assignments

Patients were categorized according to their genetic mutations: Large deletions (equal to or larger than one exon length), large duplications (equal to or larger than one exon length), and small mutations (including small deletions/insertions, single base-pair mutations such as missense/nonsense mutations and splice site mutations). For DMD mutation studies, single- or multi-exon out-of-frame deletions and duplications were confirmed using multiplex ligation-dependent probe amplification; then, second-generation sequencing was performed to identify small mutations. The genetic mutation results were confirmed by submission of a genetic report to the Duchenne Registry genetic counselors. Large deletions were further divided into deletions amenable to exon-skipping therapies at the exon 44, 45, 51, and 53 sites and to a group with exon 3–7 deletions. A patient with a single exon 52 deletion, amenable to skipping of both exon 51 and 53, was assigned to the “deletions amenable to exon 53 skipping” subgroup because of the emergence of golodirsen and viltolarsen treatments. Large deletions that could not be assigned to the aforementioned subgroups were included in the “other deletions” subgroup.

### Monitoring the clinical progression of different genotypes

Participants were evaluated at outpatient visits every year. Those who had difficulty attending outpatient visits underwent follow-up by telephone or e-mail. We measured clinical progression by recording the age at LOA and the age at the time of death (length of survival). LOA was defined as a need for continuous wheelchair use. We collected these data from the patients' guardians during regular follow-ups.

### Glucocorticoid treatments and clinical responses

In China, GC treatments for DMD usually start when the patient is between the age of 4 and 6 years before the rapid decline phase starts. Participants aged 5 years or older were assigned into three groups according to their exposure to GC: The GC-treated group, including those who received continuous GC treatment for 12 months or longer; GC-naïve group, including those who never received GC treatment or were only treated for less than 1 month; Intermittent group, including those with GC exposure that lasted longer than one month but less than 12 months. The patients in the GC group were further grouped into those who received prednisone/prednisolone and those who received deflazacort. Patients who switched between deflazacort and prednisone/prednisolone were assigned according to which treatment lasted longer. Clinical responses to these treatments among the different genotypes were measured by the age at LOA and length of survival.

### Statistical analyses

Time-to-event analyses of LOA were performed to analyze the differences between groups with age (years) as the time variable and LOA as the event. The median age at LOA and corresponding 95% confidence intervals (CIs) were estimated by plotting empirical Kaplan–Meier curves for each group defined by the mutation type and by GC treatment administered while the patient was ambulatory. Cox proportional hazard models were used to estimate and compare the age-related risks of LOA. Covariates, including DMD mutations and GC drug (deflazacort or prednisone/prednisolone) treatments, were recorded. The significance level was set at p < 0.05. All analyses were conducted using the SPSS software package (SPSS 20 Inc., Chicago, IL, USA).

## Data Availability

The datasets used during the current study are available from the corresponding author on reasonable request.

## Abbreviations

BMD: Becker muscular dystrophy
CGDR: Chinese Genetic Disease Registry
CI: Confidence intervals
CK: Creatine kinase
DMD: Duchenne muscular dystrophy
HR: Hazard ratio
LOA: Loss of ambulation
SPSS: SPSS software
